# Diet Supplemented with Antioxidant and Anti-Inflammatory Probiotics Improves Sperm Quality after Only One Spermatogenic Cycle in Zebrafish Model

**DOI:** 10.3390/nu11040843

**Published:** 2019-04-13

**Authors:** David G. Valcarce, Marta F. Riesco, Juan M. Martínez-Vázquez, Vanesa Robles

**Affiliations:** 1 IEO, Spanish Institute of Oceanography, Planta de Cultivos El Bocal, Barrio Corbanera, 39012 Monte, Santander, Spain; dgvalcarce@gmail.com (D.G.V.); riesco.mf@gmail.com (M.F.R.); juanma.martinez@ieo.es (J.M.M.-V.); 2 MODCELL GROUP, Department of Molecular Biology, Universidad de León, 24071 León, Spain

**Keywords:** sperm quality, probiotics, zebrafish, motility, behavior

## Abstract

Infertility is a medical concern worldwide and could also have economic consequences in farmed animals. Developing an efficient diet supplement with immediate effects on sperm quality is a promising tool for human reproduction and for domesticated animal species. This study aims at elucidating the effect of a short-time probiotic supplementation consisting of a mixture of two probiotic bacteria with proven antioxidant and anti-inflammatory activities on zebrafish sperm quality and male behavior. For this purpose, three homogeneous groups of males in terms of motility (<60%) were established. The control group was fed with a normal standard diet. The other received supplements: One group (vehicle control) was fed with maltodextrin and the other received a probiotic preparation based on a mixture (1:1) of *Lactobacillus rhamnosus* CECT8361 and *Bifidobacterium longum* CECT7347. The feeding regime was 21 days corresponding with a single spermatogenesis in zebrafish. The preparation did not modify animal weight, positively affected the number of fluent males, increased sperm concentration, total motility, progressive motility, and fast spermatozoa subpopulations. Moreover, the animals fed with the supplement showed different behavior patterns compared to control groups. Our results suggest a diet-related modulation on the exploration activity indicating a lower stress-like conduct. The studied formulation described here should be considered as advantageous in male reproductive biotechnology.

## 1. Introduction

Infertility is a highly ubiquitous global health problem. It has been recognized as a public health issue worldwide by the World Health Organization (WHO) and is predicted to affect 9% of the world population on average [1]. Altered production of functional and motile spermatozoa is a causal factor in up to 70% of infertility cases [2]. Sperm concentration has reduced by half in Western countries in the last four decades, without evidence of improvement [3]. Moreover, a high proportion of young men have sperm counts below the fertile threshold [4]. A variety of environmental factors may be contributing to reduced semen quality [4]. One of them is diet [5]. For example, there exists vast evidence regarding the adverse consequences of high-fat diets on male reproductive success [6]. Describing the dietary factors that can influence male fertility potential is of high interest. Nowadays, there is strong and consistent evidence about antioxidants as essential factors for sperm defense [7]. Selenium, vitamin E, vitamin C, folate, carotenoids, zinc, or carnitine are antioxidants naturally found in semen samples. The main function of these molecules is helping to overcome reactive oxygen species (ROS) production from free radicals [8]. Since subfertile men have been identified as having lower levels of these scavengers in their semen [9], they have become an object of study for reproductive biologists [10].

Probiotics are living microorganisms that improve animal health status when integrated in the diet [11]. These microorganisms act by balancing the gut microbiota, regulating the intestinal transit, modulating intestinal villi, and protecting nutrient digestion and absorption. The intestinal microbiome is a complex ecosystem, which provides numerous crucial functions to the host organism [12]. During the last decades, gut microbiota (16S rRNA surveys are used to taxonomically identify the microorganisms in the environment [13]) has emerged as a key factor which regulates host metabolism and different gut microbiome phenotypes (the genes and genomes of the microbiota, as well as the products of the microbiota and the host environment [13]) have been associated with diseases [14]. Therefore, being able to regulate intestinal microbiota is of huge interest for scientists due to the potential implications in several fields of knowledge. To date, a myriad of species from *Bacillus*, *Enterococcus*, *Lactococcus*, *Streptococcus*, *Bifidobacterium*, or *Lactobacillus* have been used as probiotics [15], with the last two genera being the most used for this purpose [16].

Nowadays, zebrafish (*Danio rerio*) is accepted by the scientific community as a vertebrate model for the study of genetics, development, and diseases among others [17,18]. This teleost is also a good model for probiotic-related experiments [19] since zebrafish microbiota is comparable to that of human as well as gut colonization [20,21]. The aim of this study was to provide, by in vivo experimentation, new insights into the potential positive effects of probiotics on male reproductive biology. The finding of new ways to increase sperm quality would be useful for clinical protocols and the possible applications derived from the potential beneficial effects of probiotics on reproductive biology will be of interest not only in human reproduction, but also in animal production where nutrition is a key element.

Our hypothesis is that probiotics during a single spermatogenesis cycle can improve sperm quality as well as animal welfare. In order to validate this hypothesis, in this work we use zebrafish since it is an optimal model for reproductive biology because of its easy reproduction, low-cost maintenance, and fast cystic spermatogenesis (21 days) [22]. To verify whether a short-term exposure to probiotics has effects on sperm quality and animal behaviour, we exposed zebrafish adult males with different initial sperm quality to a multistrain probiotic combination containing two previously described bacteria with antioxidant and anti-inflammatory activities: *Lactobacillus rhamnosus* CECT8361 and *Bifidobacterium longum* CECT7347 [23,24].

## 2. Materials and Methods 

### 2.1. Ethics Statement

The institutional Animal Care and Use Committee at the Marine Culture Plant El Bocal of the Spanish Institute of Oceanography in Santander (Spain) approved the experimental design and all protocols and procedures including animals (PI-10-16). All animals were manipulated in accordance with the Guidelines of the European Union Council (86/609/EU, modified by 2010/62/EU), following Spanish regulations (RD/1201/2005, abrogated by RD/2013) for the use of laboratory animals.

### 2.2. Animals

Wild-type zebrafish (Ab strain) were housed in the Marine Culture Plant El Bocal zebrafish platform of the Spanish Institute of Oceanography in Santander (Spain). Fish were bred and maintained according to standard protocols. In all trials, males were anesthetized in 110 mg/L buffered tricaine methane sulfonate (MS222). All efforts were made to reduce suffering and a humane endpoint was applied with a lethal dose of anesthetic if fish reached a moribund state.

### 2.3. Visible Implant Elastomer Tags (VIE) Tagging

**Figure 1:**
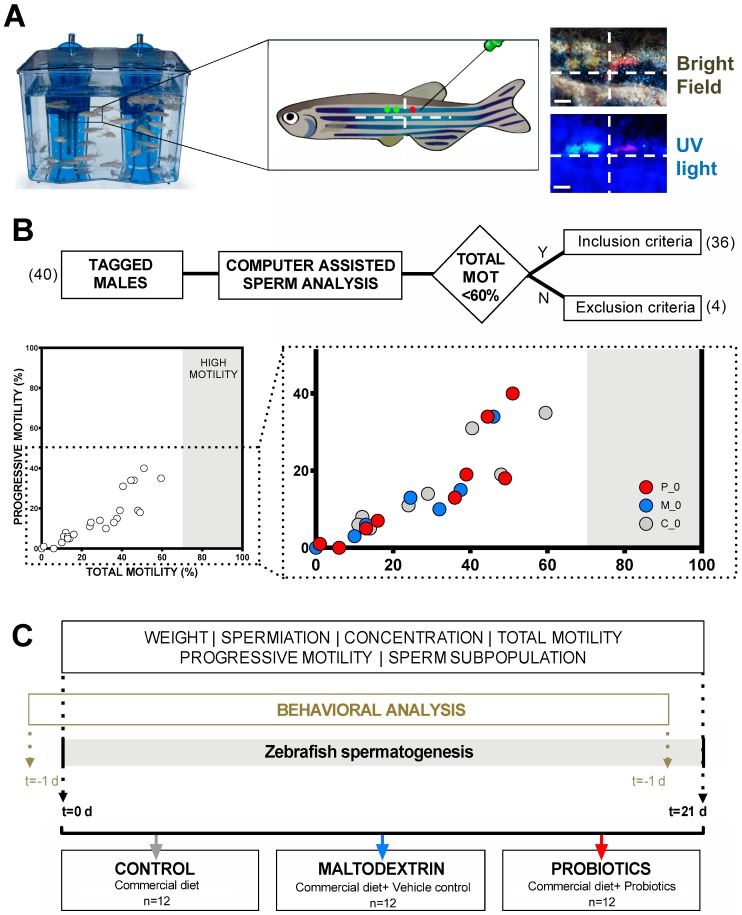
Study design summary. (**A**) Animals participating in the experiment (*n* = 40) were tracked with fluorescent visible implant elastomers. Each male carried a unique code visible under white and UV light. (**B**) Only males that reached the inclusion criteria described in the flowchart (*n* = 36) were selected for creating homogeneous experimental groups in terms of total motility. (**C**) Each group (*n* = 12) received a different diet during 21 days corresponding to a *Danio rerio* spermatogenesis cycle. “C”, “M”, and “P” refer to the experimental groups: Control, maltodextrin, and probiotics, respectively.

VIE tags were prepared following manufacturer’s indications adapted for the minimal volume (Northwest Marine Technology, Shaw Island, WA, USA). Green and red fluorescent elastomers (viscoelastic polymers) were used in the experiment. A code combining colors, number of tags, and positions (taking as reference the anteroposterior and dorsoventral axis) was generated and individually associated to a specific male in the zebrafish colony (Figure 1A). An expert hand injected small amounts of elastomers (dot shaped) in each anesthetized male. After injection, tag retention and injury evaluation (not registered) was evaluated in the recovery tanks. Health status was checked daily.

### 2.4. Inclusion Criteria, Experimental Group Definition, Study Design, and Feeding Regimes

Adult zebrafish males were anesthetized and sperm samples were collected and evaluated with a CASA (computed assisted sperm analysis) system (see below for procedure). Only males showing a total motility under 60% were selected for the experiment (Figure 1B). The inclusion criterion was established this way to analyze the effect of the probiotic strains mixture on diverse quality sperm samples. The cutoff value of 60% was chosen with the aim that there would be an improvement margin in the samples after the treatment. Semen samples over 60% can be considered acceptable samples in terms of motility. Males reaching the inclusion criteria were used to generate three homogeneous groups (*n* = 12) in terms of motility (Figure 1B). Each group had a different feeding regime: (1) The control group “CTRL” ingested only a commercial diet; (2) the vehicle control group “MALTO” received the commercial diet and two doses of 0.11 g of maltodextrin; and (3) the experimental group “PROBIO” received the commercial diet and a probiotic treatment consisting of a daily 10^9^ Colony Forming Units (CFU) mixture (1:1) of lyophilized *L. rhamnosus* CECT8361 and *B. longum* CECT7347 strains carried in 0.22 g of maltodextrin. Strains were kindly provided by the company Biopolis S.L. (Valencia, Spain) and the commercial diet provided to all animals within the experiment twice a day (55% min. crude protein, 15% min. crude fat, 1.5% max. crude fiber, and 12% max. moisture) was purchased from Aquatic Animals (Apopka, FL, USA). In order to guarantee the ingestion, supplements (vehicle or probiotic mixture) were provided to experimental males in rearing water 30 min before each routine feeding. All experimental groups were held under the same conditions during all experiments. The experiment was replicated three times, including four males per experimental group each time (final population for each experimental group, *n* = 12). Sperm analysis was performed at t = 0 days and t = 21 days of each experimental replicate. Feeding regimes were maintained during the 21 days according to a spermatogenesis cycle in the species (Figure 1C).

### 2.5. BiometricAnalysis

At day 0 and day 21, fish weight was determined using a microbalance (Mettler MT5, Mettler Toledo, Spain).

### 2.6. Sperm Sampling

At 0 days and at 21 days, semen was collected approximately 1h after the lights of the zebrafish facility were turned on. Each fish was identified by checking its VIE tag code and after that, they were anesthetized one by one. Once the absence of reflexes was corroborated, the animals were gently located on a sponge, the surrounding area of the urogenital pore was dried and sperm collection was performed by abdominal massage using glass flat forceps as tools to smoothly press both sides according to routine protocols. Ejaculates were collected with a micropipette and diluted in 10 µL of buffered Hank’s solution (0.137 M NaCl; 5.4 mM KCl; 0.25 mM Na_2_HPO_4_; 0.44 mM KH_2_PO_4_; 1.3 mM CaCl_2_; 1.0 mM MgSO_4_; 4.2 mM NaHCO_3_). The diluted samples were stored at 22ºC until analysis (5 min).

### 2.7. CASA Sperm Analysis

The activation of motility was performed by diluting 1 µL of sperm with 9 µL of system water (~300 mOsm/L) at 28 °C. Sperm motility, kinetics, and concentration were analyzed using a CASA system with ISAS software (ISAS, PROiSERR+D, S.L. Spain). Activated sperm was loaded into a Makler counting chamber (10 µm depth; Sefi Medical Instruments, Haifa, Israel). The CASA system consisted of a tri-ocular optical phase-contrast Nikon Eclipse Ts2R microscope (Nikon, Tokyo, Japan) using a 10× objective equipped with Basler A312fc digital camera (Basler Vision Technologies, Ahrensburg, Germany). The ISAS software was used with specific settings for fish spermatozoa (1 µm^2^ < particle area < 20 µm^2^; cell description according to VCL (curvilinear velocity): 10 µm/s < slow < 45 µm/s < medium < 100 µm/s < fast); and it rendered the following parameters: (1) Concentration; (2) percentage of motile spermatozoa (MOT,%); (3) percentage of progressive spermatozoa (P-MOT,%) defined as the percentage of spermatozoa which swim forward in 80% of a straight line; (4) curvilinear velocity (VCL, µm/s) defined as the time per average velocity of a sperm head along its actual curvilinear trajectory; (5) average path velocity (VAP, µm/s) defined as the time per average velocity of a sperm head along its spatial average trajectory; (6) straight-line velocity (VSL, µm/s) defined as the time per average velocity of a sperm head along the straight line between its first-detected position and its last position; (7) linearity of the curvilinear path (LIN, %), expressed as VSL/VCL; (8) straightness (STR,%) defined as VSL/VAP; (9) wobble (WOB,%) expressed as VAP/VCL; (10) amplitude of the lateral head displacement (ALH, µm); and (11) beat cross frequency (BCF, Hz) based on VCL crossing VAP per second. Motility parameters were evaluated at 15 s after activation to avoid drifting and to corroborate that all samples were measured at an exact equal time post activation. At least, 200 spermatozoa were analyzed for each sample. Three fields per sample were evaluated. If samples reported very low concentrations, more than three fields were captured.

### 2.8. Behavior Analysis

To test the exploratory behavior of the animals, we used a novel tank test (NTT), which evokes motivational conflict between the “protective” diving behavior and subsequent vertical examination following established procedures. Briefly, each animal was individually placed in the evaluation arena (20 cm (*x*) × 18 cm (*y*) × 8 cm (*z*); swimming volume: 3.5 L). Males were let to acclimate to the new environment for 3 min and right after animal behaviour was filmed (1920 × 1080 px) for 3 min. Individual male swimming activity was monitored using the free digital video tracking software Tracker (physlets.org/tracker/). The actual position of the animal was manually located every 20 frames to avoid possible inaccuracies of the automatic option of the software. Then, each resulting track was evaluated using a virtual grid pattern with upper and lower subareas in order to allow quantification and comparison between experimental groups. For each animal we quantified two estimates of exploratory behavior: Number of crossings between the upper-half subarea and the lower one and the percentage of time spent in the upper half of the arena.

### 2.9. Data Analysis

Results are expressed as the mean ± standard error. Statistical differences between mean values of each variable at 0 and 21 days were determined using a t-Student test for correlated variables for normally distributed variables or a Wilcoxon test for paired samples for non-parametric variables. A principal component analysis was performed for the set of observed variables for CASA parameters. All statistical analysis were performed using Prism 8 (GraphPad Software, San Diego, CA, USA) and SPSS V. 22 (SPSS Inc., Chicago, IL, USA). *P*-values < 0.0500 were considered statistically significant.

## 3. Results

### 3.1. Effects of Probiotic Mixture Supplementation on Total Body Weight and Spermiation Capacity

**Figure 2:**
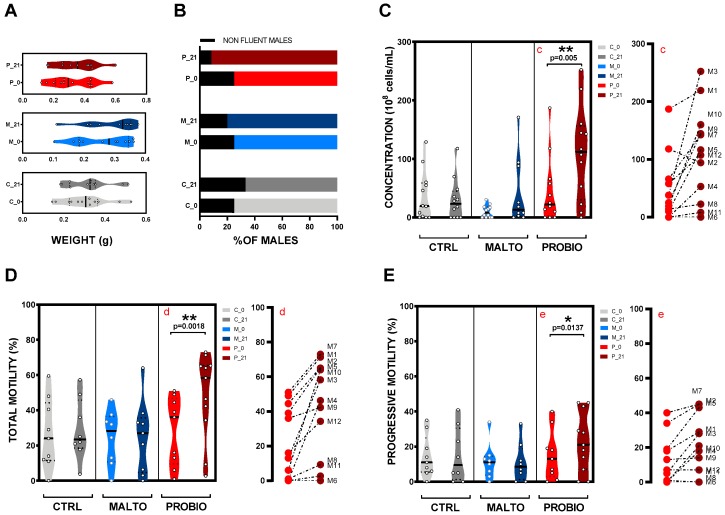
Probiotic mixture supplementation effects on zebrafish males and sperm quality after a cycle of spermatogenesis exposure. (**A**) None of the studied diets modified male total body weight. (**B**) Spermiation ability of studied males before and after the experiment. (**C**) Concentration, (**D**) total motility, and (**E**) progressive motility at 0 and 21 days obtained for each experimental group represented with violin graphs. “C”, “M”, and “P” refer to the experimental groups: Control, maltodextrin, and probiotics respectively. Furthermore, “c”, “d”, and “e” are before–after graphs for the PROBIO group where “M#” indicates the number of the male. Asterisks show statistically significant differences: * (*p* < 0.0500), ** (*p* < 0.0100).

In order to investigate the effects of probiotic supplementation in male zebrafish on growth parameters, we weighed the animals included in the experiment at t = 0 days and t = 1 days (Figure 1). As expected, taking into account the short temporal frame of our experiment, our analysis revealed no statistical differences (*p* > 0.0500) at day 21 in any of the experimental groups: Control (C; CTRL); maltodextrin, the vehicle control (M; MALTO); or probiotic-fed (P; PROBIO) (Figure 2A; Supplementary File Table S1). These data provide confidence about the suitability of maltodextrin as a carrier in our probiotic-fed group.

As a first general parameter regarding spermatogenesis, we focused our attention on spermiation ability. As a result of a correct spermatogenesis, mature spermatozoa are released from cysts into the lumen of the tubules and therefore ejaculated. At day 0, we arranged population homogeneously with three (25%; CTRL), four (33%; MALTO), and three (25%; PROBIO) non-spermiating males in each group. After a single cycle of spermatogenesis (21 days) the non-fluent male percentage changed as follows: four (33%; CTRL), two (20%; MALTO), and one (8.33%; PROBIO). Interestingly, a spermiation modulation was suggested with this data in males supplemented with bacteria strains (PROBIO). In this group, only one male did not provide sperm at day 21 sampling (Figure 2B).

### 3.2. Effects of the Probiotic Mixture on Concentration, Total Sperm Motility, and Progressive Motility

To study the effects of the ingested probiotic strains on a single cycle of spermatogenesis, we studied individually, using VIE tagging for male tracking, the sperm samples in terms of concentration, total motility, and progressive motility. At day 0, all males included in this study presented an initial total motility below 60%. Groups were created including a wide range of sperm motility values from 0% motility to the 60% threshold (Figure 1B). Results regarding sperm concentration, total motility, and progressive motility are presented in Figure 2.

Bacteria ingestion strongly modified (*p* = 0.0050) sperm count (10^8^ cells/mL; mean ± s.e.) in the PROBIO group after 21 days of supplementation (Figure 2C; Table S2). The mean value for this variable increased from 44.58 ± 16.40 to 110.10 ± 23.13. Controls reported lower concentrations at day 21: 30.19 ± 10.15 (CTRL) and 41.57 ± 18.16 (MALTO), respectively.

Regarding total motility (%; mean ± s.e.), controls showed similar values (*p* > 0.0500) before and after the experiment. Mean values were: 26.44 ± 6.528 (day 0) vs. 28.97 ±6.194 (day 21) for the CTRL group and 24.56 ± 5.53 (day 0) vs. 24.97 ±6.77 (day 21) for the MALTO group (Figure 2D; Table S2). In contrast, the animals with a feeding regime supplemented with probiotics (PROBIO) revealed a substantial rise (*p* = 0.0018) in total motility from 28.39 ± 6.46 (day 0) to 48.36 ± 7.32 (day 21). When a detailed individual evaluation of data was performed, results showed strong increments (>40%) in 11 of 12 studied males. Four fish increased their sperm motility more than 100%: M3, M8, M10, and M12 (Figure 2D.d). These results evidence a very strong positive effect of probiotic ingestion on zebrafish sperm quality. Please find individual before–after graphs for CTRL and MALTO groups in Figure S1.

Moreover, and concomitantly with total motility, progressive motility (P-MOT) was also significantly raised (*p* = 0.0137) after 21 days in the PROBIO group from 15.22% ± 4.71% to 22.73% ± 5.09% (Figure 2E; Table S2). P-MOT is another key parameter in sperm quality since it influences fertilization success, and it is a focus of attention in zebrafish research [25,26]. In our experiment, we considered progressive those cell in which swimming track was forwards in 80% of a straight line.

### 3.3. Effects of the Probiotic Mixture on Sperm Kinematic Parameters

**Figure 3:**
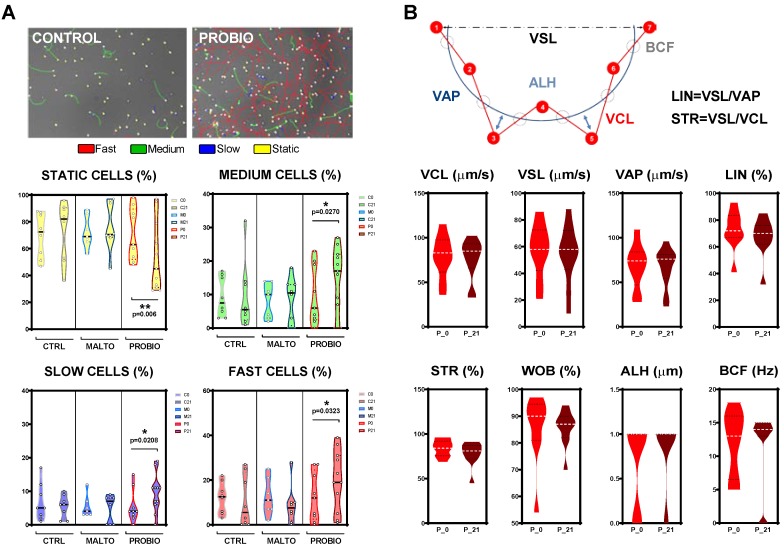
Probiotic mixture supplementation effects on zebrafish sperm kinematics after a cycle of spermatogenesis exposure. (**A**) Sperm subpopulations within the motile population according to speed parameters before and after the experiment for each group. (**B**) Detailed sperm kinetics rendered by the CASA system for the PROBIO group. Asterisks show statistically significant differences; * (*p* < 0.0500). Abbreviations: VCL—curvilinear velocity, VSL—straight line velocity, VAP—average path velocity, LIN—linearity of the curvilinear path, STR—straightness, WOB—wobble, ALH—amplitude of the lateral head displacement, BCF—beat cross frequency.

We studied sperm kinetics in depth since it has been reported that external-fertilizing fish have the highest known intensity of sperm competition of any external fertilizing vertebrates. Thus, the presence of fast subpopulations within the motile cells seems to be an advantage and, therefore, it can be considered a parameter of sperm quality. In this experiment subpopulations were clustered in terms of VCL. Four groups were established and set up in the CASA software: Static, slow, medium, and fast according to the following thresholds: 10 µm/s < slow < 45 µm/s< medium < 100 µm/s < fast. Interestingly, only the PROBIO group showed statistical differences at the end of the experiment (Figure 3A) in the four subpopulations: Static (*p* = 0.006), slow (*p* = 0.0208), medium (*p* = 0.0270), and fast (*p* = 0.0323) cells.

Concerning kinematic parameters, there was no overall difference (*p* > 0.0500) in sperm velocities (VCL, VSL, VAP), linearity (LIN), straightness (STR), wobble (WOB), amplitude of the lateral head displacement (ALH), or beat cross frequency (BCF). Figure 3B shows these results for the PROBIO experimental group. These results suggest that the effect of probiotic bacteria do not fine tune zebrafish sperm kinetics. PCA results can be found in Figure S2.

### 3.4. Effects of the Probiotic Mixture on Male Behavior

**Figure 4:**
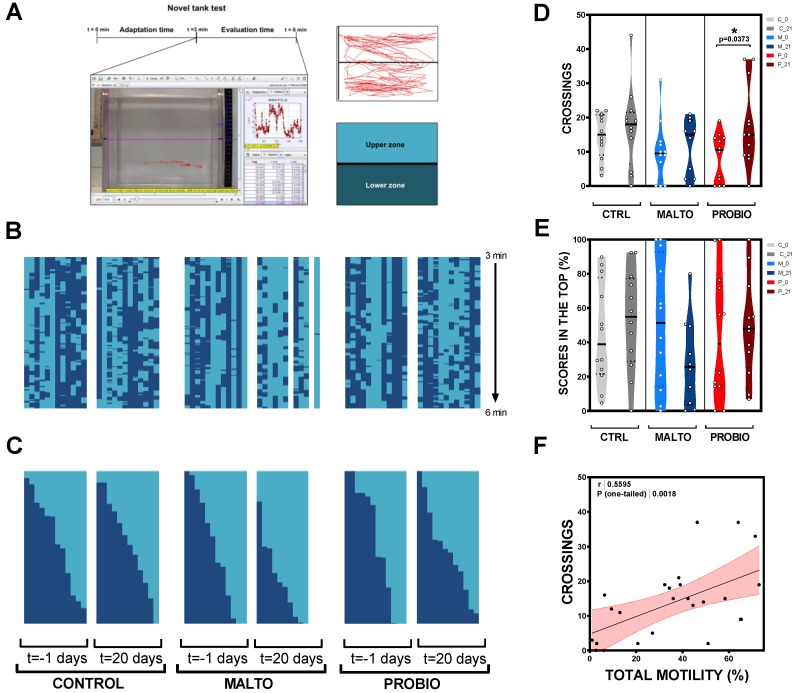
Probiotic bacteria ingestion modulates male behavior in zebrafish. (**A**) Schematic representation of the novel tank test (NTT). (**B**) Individual summaries of the track for each animal in the experiment at 0 and 21 days. (**C**) Organized summaries from higher to lower scores in the lower subarea. (**D**) Comparison of the scores registered in the upper zone before and after treatment. (**E**) Comparison of the number of crossings between the two subareas of the arena at 0 and 21 days. (**F**) Correlation between “number of crossings” and “total motility” variables integrating data from the three experimental groups after probiotic treatment. Asterisk shows statistically significant differences * (*p* < 0.0500).

After three minutes of adaptation time to a new environment, tracking analysis were performed to evaluate the anxiety status of the fish. Quantification of the novel tank test (NTT) was carried out attending to two variables: (1) The percentage of positions scored in each of the two virtual zones (upper and lower) of the novel tank and (2) the number of crossings from one to another (Figure 4A). The novel tank test (NTT) is the conceptual equivalent of the rodent open field (OF) paradigm; NTT induces motivational conflict between the “defensive” diving behavior and following vertical exploration [27,28]. Each male was analyzed at the beginning and at the end of the experiment a day before sperm squeezing (*t* = −1 day and *t* = 20 days). Summaries of each animal behavior were created for easier evaluation (Figure 4B). As can be checked in Figure 4C,E, there was a non-statistical (*p* > 0.0500) trend toward PROBIO fish spending more time in the top of the tank, close to the 50% in mean values, at day 21 (47.92% ± 8.37%) compared to day 0 (39.25% ± 4.71%). The number-of-crossings evaluation revealed again significant differences (*p* = 0.0373) only in the PROBIO population doubling the mean values of the variable (Figure 4D; Table S3). Moreover, a moderate correlation (*p* = 0.0018; *r* = 0.5595) between fish behavior and total motility of squeezed ejaculates was observed (Figure 4F).

## 4. Discussion

Nutrition could have a positive or negative impact on reproduction. Nowadays, decrease in sperm quality could be considered a global health problem. Indeed, asthenozoospermia is one of the male subfertility pathologies described by the WHO (2010) as a condition in which the percentage of progressively motile sperm is abnormally low [29]. Since the development and optimization during the last decades of the artificial reproductive technologies (ARTs): Intracytoplasmic sperm injection (ICSI), ovarian stimulation, intrauterine insemination (IUI), or in vitro fertilization (IVF) many infertile couples have found a solution to conceive. Although they have become a major worldwide focus of attention, these techniques are expensive and invasive. Possible clinical approaches may include antioxidant ingestion as a preliminary or concomitant treatment to reproductive techniques to improve fertility outcomes.

The definition of oxidative stress (OS) is the overabundance of reactive oxygen species (ROS) or a deficiency of antioxidants [30]. The imbalance produced by ROS causes cell damage. The deleterious effects of this damage on spermatozoa have been known since the 80 s [31]. There exists evidence regarding the need of certain amounts of ROS for normal sperm functions of both in mammals [32] or teleost [33] mainly produced by the mitochondria. However, excessive quantities become pathophysiological and lead to DNA damage and even apoptosis [7]. Endogenous or exogenous factors may be the cause of high levels of ROS. The most common exogenous causes of OS are obesity, smoking, environmental contaminants, alcohol intake, and malnutrition [9]. Natural antioxidants can scavenge ROS, inactivate them, and repair the cellular damage [34]. Spermatozoa, due to their high specialization, do not present cytoplasm after spermatogenesis and they depend on seminal plasma, which is rich in antioxidants [35]. Moreover, in spermatozoa, polyunsaturated fatty acids (a highly oxidizable substrate) enrich the cell membrane, provoking a high vulnerability to lipid peroxidation from ROS both in mammals [5] and teleost [36]. Oxidative damage affects the sperm flexibility and therefore motility, which is the excellence parameter to assess sperm quality. The spermatozoal heightened vulnerability to OS has caused enormous interest in the role of diet antioxidants in the management of infertile men [7].

Nowadays, the probiotic market is increasing globally as a cheap and well accepted (by the consumers) supplement source all around the world. There exists an increase in the demand of these kinds of products to improve health or prevent human illness. The developing observation that the gut microbiota plays a central role in regulating the host’s physiology has supported the significance of the probiotic concept. The modulation of the intestinal microbiota composition has been proposed as one of the main mechanism of probiotic activity [37]. In a previous study, our group reported that the effects of a commercial probiotic diet supplement (Bactocell^®^, Lallemand Animal Nutrition S.A., Blagnac, France), containing a lactic acid bacteria strain (*Pediococcus acidilactici*) improved molecular sperm quality markers in zebrafish testicular cells after a short period (10 days) [38], providing initial data regarding the potential use of probiotic supplementation on zebrafish male reproductive performance. In the present study, the ingestion of a supplement containing probiotic strains on a single cycle spermatogenesis evidenced a positive effect of the host’s sperm quality after a single cycle of spermatogenesis in zebrafish model. Specifically, the present study was undertaken to evaluate the combined effects of two strains: *L. rhamnosus* CECT8361 and *B. longum* CECT7347. These strains were selected because they belong to the most-used genera as probiotics nowadays [16] and they have been previously described as strains with antioxidant activity [23]. Additionally, *B. longum* CECT7347 has been assigned with anti-inflammatory activity. This strain has been described to reduce the inflammatory effects of the dysbiotic intestinal microbiota of individuals with coeliac disease on peripheral blood mononuclear cells partially via the induction of IL-10 production [24,39]. *B. longum* CECT7347 has also been demonstrated to decrease the cytotoxic and inflammatory effects of gliadin peptides on epithelial cell in vitro degradation [40,41]. Furthermore, in the gliadin-induced enteropathy animal model, this strain has been shown to reduce the peripheral CD4^+^ T cells, rise IL-10, and shrink TNF-α production [42]. The other strain used in this experiment belongs to the *Lactobacillus* genera, which has been repeatedly shown as the predominant bacteria in the semen, accompanied by a flexible composition of other taxa [43,44,45]. *L. rhamnosus* species has been described as a highly adhesive bacteria in zebrafish [21]. The specific mechanism by which these bacterial strains are modifying fish behaviour and sperm quality in the present study is unclear. It is known that the ingestion of antioxidants can improve sperm motility [7], but the anti-inflammatory properties of *B. longum* CECT7347 could not be ignored and cannot be separately evaluated in our study. In fact, in humans, it has been reported that the ingestion of a combination of the two strains *L. rhamnosus* and *B. longum* modulated the gut microbiota composition, leading to a significant reduction of potentially harmful bacteria and an increase of beneficial ones [46]. Indeed, the combination of specific bacterial strains belonging to these two species can act in optimal synergy for restoring the intestinal balance [47] even better than individually [48].

In the present study, it was demonstrated that in the zebrafish model, males fed with the probiotic formulation increased sperm quality. In particular, in terms of sperm counts, 11 of the 12 males within the PROBIO group showed an improvement in concentration (Figure 2C), independent of initial values after 21 days of ingestion. The individual track of animals was available thanks to the use of VIE tagging. This technique is starting to spread among facilities since this inert, non-immunogenic polymer is useful for many purposes. The results achieved by this in vivo study clearly showed that the ingestion of the combination of *L. rhamnosus* CECT8361 and *B. longum* CECT7347 increased the percentage of motile cells after a single cycle of spermatogenesis (Figure 2D). After 21 days of exposure, a clear induction of total motility was found in all males within the PROBIO group, contrary to control cohorts (Figure 2D). These results are in line with our previous observations in asthenozoospermic human samples [23] on the ability of the same couple of probiotics reporting an increase of total motility after treatment. In the current study, total motility improved with a 1.7-fold change. Concomitantly to the increment in total motility values, progressive motile cells were also improved (Figure 2D), although the fold-change before and after the probiotics ingestion was lower. In our results, the increment of total motility was also accompanied by a modulation of sperm subpopulations within the motile population (Figure 3). All slow, medium, and fast motile cell populations were increased after 21 days in the probiotic-fed animals contrary to diet-controlled and vehicle-fed ones (Figure 3). Interestingly, the spermatozoa kinetics did not show differences after the experimental time indicating that probiotic bacteria are not able to alter these parameters in the zebrafish model. Altogether, the capability of *L. rhamnosus* CECT8361 and *B. longum* CECT7347 to modulate sperm quality was remarkably corroborated. Our results regarding sperm quality improvement are further supported by some studies [49,50] involving the use of probiotics in other animal models, which reported a potentially positive effect of probiotics in terms of sperm quality parameters. In particular, Dardmeh and colleagues [50] demonstrated that *L. rhamnosus* PB01 (DSM 14870) may have an effect on weight after eight weeks of treatment as well as a modulation of sperm kinetics and hormone levels in mice with diet-induced obesity. It has also been suggested that the use of *Bacillus amyloliquefaciens* TOA5001 as a probiotic has potential positive effect on broiler breeders, since the strain was able to increase sperm count and sperm viability after six weeks of treatment [49].

The scientific community is starting to elucidate the mechanisms provoking these beneficial effects of probiotic ingestion on sperm quality. Recently, Kelton Tremellen has published a novel theory [51], the GELDING theory (Gut Endotoxin Leading to a Decline IN Gonadal function) in which it is postulated that “the trans-mucosal passage of bacterial lipopolysaccharide from the gut lumen into the circulation is a key inflammatory trigger underlying male hypogonadism”. The author has also linked the theory to a described positive effect of probiotics on human sperm samples from infertile patients [52]. This new and interesting theory is remarkable after analysing our results. The synergy between antioxidant and anti-inflammatory properties of the two bacteria used in the present study may explain the registered improvement in zebrafish sperm quality. According to Tremellen’s theory, this assumption may be accepted.

Noticeably, in the present study, we offer evidence that in adult male zebrafish, short ingestion of probiotics modulates behavior pattern (Figure 4). Zebrafish is an interesting model organism to investigate behavior. Founded on geotaxis—an innate escape “diving” behavior of fish in novel environments—the novel tank test (NTT) has long been used to analyze adult zebrafish behaviors [53] and drug responses [54]. Adult zebrafish initially spend more time at the lower part of the tank when they are exposed to a novel environment. Concomitantly, they reduce “top” swimming and reveal more unpredictable movements and show freezing/immobility events [55]. Subsequent, because of habituation to the NTT novelty, animals gradually explore the top area (theoretically less safe for zebrafish in their wild habitats due to predator risk) [28]. Although our results did not report statistically significant differences in the number of scores in the upper subarea of the novel arena before and after probiotic administration, the number of crossing between the bottom and the upper area revealed differences (Figure 4). These results suggest that *L. rhamnosus* CECT8361 and *B. longum* CECT7347 modulate the exploration activity of the males after only 21 days of exposure showing a lower stress-like conduct. The microbiota signals to the central nervous system (CNS) via several potential pathways [56]. Probable mechanisms of communication embrace production of various metabolites that cross the intestinal barrier into the circulatory system, and/or microbe-derived metabolites that can signal through the immune system [57]. Moreover afferent pathways of the vagus nerve from the enteric nervous system (ENS) to the CNS have been associated as a key route of communication concerning the microbiota and CNS [58]. Our results are in accordance with a number of recent findings reporting that the use of various *Lactobacillus* and/or *Bifidobacterium* strains can lighten anxiety-and depressive-like behavior and alleviate stress responses in animal models [58,59,60,61].

## 5. Conclusions

In conclusion, our study showed that 21 days of treatment (a spermatogenesis cycle) with a probiotic mixture with described antioxidant and anti-inflammatory activities significantly improved zebrafish sperm quality and increased the number of fluent males. These data highlight the promising use of this probiotic mixture to improve reproductive performance in different quality sperm samples by increasing sperm total motility, progressive motility, concentration, and fast sperm populations. Furthermore, behavior analysis revealed a modulation in probiotics-fed males suggesting a lower anxiety-like pattern, which may be correlated with the improvement of sperm quality parameters in this model. Considering the simplicity and economical effectiveness of the studied multistrain product, the results presented here strengthen the potential use of this preparation in male reproductive biotechnology, which may be useful in the aquaculture industry and reproductive biology fields.

## Supplementary Materials

The following are available online at https://www.mdpi.com/2072-6643/11/4/843/s1, Figure S1: Before–after graphs for (**A**) concentration, (**B**) total motility, and (**C**) progressive motility at 0 and 21 days obtained for each experimental group. “M” and “P” refers to the experimental groups: Control and maltodextrin respectively. “M#” indicates the number of the male; Figure S2: (**A**) PCA analysis for Computer Assisted Sperm Analysis (CASA) variables for probiotic-fed group. (**B**) Representation of the experimental group in a principal component plane; Table S1: Weight values (mean ± s.e.) for the groups before and after treatment; Table S2: Sperm quality values (mean ± s.e.) for the groups before and after treatment; Table S3: novel tank values for behavior estimators (mean ± s.e.) for the groups before and after treatment.

## References

[1] Boivin J., Bunting L., Collins J.A., Nygren K.G. (2007). International estimates of infertility prevalence and treatment-seeking: Potential need and demand for infertility medical care. Hum. Reprod..

[2] Agarwal A., Mulgund A., Hamada A., Chyatte M.R. (2015). A unique view on male infertility around the globe. Reprod. Biol. Endocrinol..

[3] Levine H., Jørgensen N., Martino-Andrade A., Mendiola J., Weksler-Derri D., Mindlis I., Pinotti R., Swan S.H. (2017). Temporal trends in sperm count: A systematic review and meta-regression analysis. Hum. Reprod. Update.

[4] Virtanen H.E., Jørgensen N., Toppari J. (2017). Semen quality in the 21st century. Nat. Rev. Urol..

[5] Nassan F.L., Chavarro J.E., Tanrikut C. (2018). Diet and men’s fertility: Does diet affect sperm quality?. Fertil. Steril..

[6] Crean A.J., Senior A.M. (2019). High-fat diets reduce male reproductive success in animal models: A systematic review and meta-analysis. Obes. Rev..

[7] Smits R.M., Mackenzie-Proctor R., Fleischer K., Showell M.G. (2018). Antioxidants in fertility: Impact on male and female reproductive outcomes. Fertil. Steril..

[8] Talevi R., Barbato V., Fiorentino I., Braun S., Longobardi S., Gualtieri R. (2013). Protective effects of in vitro treatment with zinc, d-aspartate and coenzyme q10 on human sperm motility, lipid peroxidation and DNA fragmentation. Reprod. Biol. Endocrinol..

[9] Tremellen K. (2008). Oxidative stress and male infertility—A clinical perspective. Hum. Reprod. Update.

[10] Cardoso J.P., Cocuzza M., Elterman D. (2019). Optimizing male fertility: Oxidative stress and the use of antioxidants. World J. Urol..

[11] Fuller R. (1989). Probiotics in man and animals. J. Appl. Bacteriol..

[12] Zmora N., Suez J., Elinav E. (2019). You are what you eat: Diet, health and the gut microbiota. Nat. Rev. Gastroenterol. Hepatol..

[13] Whiteside S.A., Razvi H., Dave S., Reid G., Burton J.P. (2015). The microbiome of the urinary tract—A role beyond infection. Nat. Rev. Urol..

[14] Hall A.B., Tolonen A.C., Xavier R.J. (2017). Human genetic variation and the gut microbiome in disease. Nat. Rev. Genet..

[15] Hill C., Guarner F., Reid G., Gibson G.R., Merenstein D.J., Pot B., Morelli L., Canani R.B., Flint H.J., Salminen S. (2014). The International Scientific Association for Probiotics and Prebiotics consensus statement on the scope and appropriate use of the term probiotic. Nat. Rev. Gastroenterol. Hepatol..

[16] Fijan S. (2014). Microorganisms with claimed probiotic properties: An overview of recent literature. Int. J. Environ. Res. Public Health..

[17] Grunwald D.J., Eisen J.S. (2002). Headwaters of the zebrafish—Emergence of a new model vertebrate. Nat. Rev. Genet..

[18] Keller E.T., Murtha J.M. (2004). The use of mature zebrafish (*Danio rerio*) as a model for human aging and disease. Comp. Biochem. Physiol. C Toxicol. Pharmacol..

[19] Carnevali O., Avella M.A., Gioacchini G. (2013). Effects of probiotic administration on zebrafish development and reproduction. Gen. Comp. Endocrinol..

[20] Gioacchini G., Giorgini E., Olivotto I., Maradonna F., Merrifield D.L., Carnevali O. (2014). The influence of probiotics on zebrafish *Danio rerio* innate immunity and hepatic stress. Zebrafish.

[21] Zhou Z., Wang W., Liu W., Gatlin D.M., Zhang Y., Yao B., Ringø E. (2012). Identification of highly-adhesive gut *Lactobacillus* strains in zebrafish (*Danio rerio*) by partial rpoB gene sequence analysis. Aquaculture.

[22] Schulz R.W., de França L.R., LeGac F., Chiarini-Garcia H., Nobrega R.H., Miura T. (2010). Spermatogenesis in fish. Gen. Comp. Endocrinol..

[23] Valcarce D.G., Genovés S., Riesco M.F., Martorell P., Herráez M.P., Ramón D., Robles V. (2017). Probiotic administration improves sperm quality in asthenozoospermic human donors. Benef. Microbes.

[24] Medina M., De Palma G., Ribes-Koninckx C., Calabuig M., Sanz Y. (2008). *Bifidobacterium* strains suppress in vitro the pro-inflammatory milieu triggered by the large intestinal microbiota of coeliac patients. J. Inflamm..

[25] Guo W., Xie B., Xiong S., Liang X., Gui J.-F., Mei J. (2017). miR-34a Regulates Sperm Motility in Zebrafish. Int. J. Mol. Sci..

[26] Sadeghi S., Pertusa J., Yaniz J.L., Nuñez J., Soler C., Silvestre M.A. (2018). Effect of different oxidative stress degrees generated by hydrogen peroxide on motility and DNA fragmentation of zebrafish (*Danio rerio*) spermatozoa. Reprod. Domest. Anim..

[27] Kalueff A.V., Stewart A., Kadri F., Dileo J., Chung K.M., Cachat J., Goodspeed J., Suciu C., Roy S., Gaikwad S. (2010). The Developing Utility of Zebrafish in Modeling Neurobehavioral Disorders. Int. J. Comp. Psychol..

[28] Kysil E.V., Meshalkina D.A., Frick E.E., Echevarria D.J., Rosemberg D.B., Maximino C., Lima M.G., Abreu M.S., Giacomini A.C., Barcellos L.J.G. (2017). Comparative Analyses of Zebrafish Anxiety-Like Behavior Using Conflict-Based Novelty Tests. Zebrafish.

[29] Cooper T.G., Noonan E., Von Eckardstein S., Auger J., Gordon Baker H.W., Behre H.M., Haugen T.B., Kruger T., Wang C., Mbizvo M.T. (2010). World Health Organization reference values for human semen characteristics. Hum. Reprod. Update.

[30] Agarwal A., Rana M., Qiu E., AlBunni H., Bui A.D., Henkel R. (2018). Role of oxidative stress, infection and inflammation in male infertility. Andrologia.

[31] Aitken R.J., Clarkson J.S. (1987). Cellular basis of defective sperm function and its association with the genesis of reactive oxygen species by human spermatozoa. J. Reprod. Fertil..

[32] Aitken J., Fisher H. (1994). Reactive oxygen species generation and human spermatozoa: The balance of benefit and risk. Bioessays.

[33] Valcarce D.G., Robles V. (2016). Effect of captivity and cryopreservation on ROS production in *Solea senegalensis* spermatozoa. Reproduction.

[34] Mirończuk-Chodakowska I., Witkowska A.M., Zujko M.E. (2018). Endogenous non-enzymatic antioxidants in the human body. Adv. Med. Sci..

[35] Zini A., de Lamirande E., Gagnon C. (1993). Reactive oxygen species in semen of infertile patients: Levels of superoxide dismutase- and catalase-like activities in seminal plasma and spermatozoa. Int. J. Androl..

[36] Cabrita E., Martínez-Páramo S., Gavaia P.J., Riesco M.F., Valcarce D.G., Sarasquete C., Herráez M.P., Robles V. (2014). Factors enhancing fish sperm quality and emerging tools for sperm analysis. Aquaculture.

[37] Hemarajata P., Versalovic J. (2013). Effects of probiotics on gut microbiota: Mechanisms of intestinal immunomodulation and neuromodulation. Ther. Adv. Gastroenterol..

[38] Valcarce D.G., Pardo M.Á., Riesco M.F., Cruz Z., Robles V. (2015). Effect of diet supplementation with a commercial probiotic containing *Pediococcus acidilactici* (Lindner, 1887) on the expression of five quality markers in zebrafish (*Danio rerio* (Hamilton, 1822)) testis. J. Appl. Ichthyol..

[39] De Palma G., Kamanova J., Cinova J., Olivares M., Drasarova H., Tuckova L., Sanz Y. (2012). Modulation of phenotypic and functional maturation of dendritic cells by intestinal bacteria and gliadin: Relevance for celiac disease. J. Leukoc. Biol..

[40] Laparra J.M., Sanz Y. (2010). Bifidobacteria inhibit the inflammatory response induced by gliadins in intestinal epithelial cells via modifications of toxic peptide generation during digestion. J. Cell. Biochem..

[41] Olivares M., Laparra M., Sanz Y. (2011). Influence of *Bifidobacterium longum* CECT 7347 and gliadin peptides on intestinal epithelial cell proteome. J. Agric. Food Chem..

[42] Laparra J.M., Olivares M., Gallina O., Sanz Y. (2012). Bifidobacterium longum CECT 7347 modulates immune responses in a gliadin-induced enteropathy animal model. PLoS ONE.

[43] Hou D., Zhou X., Zhong X., Settles M.L., Herring J., Wang L., Abdo Z., Forney L.J., Xu C. (2013). Microbiota of the seminal fluid from healthy and infertile men. Fertil. Steril..

[44] Weng S.-L., Chiu C.-M., Lin F.-M., Huang W.-C., Liang C., Yang T., Yang T.-L., Liu C.-Y., Wu W.-Y., Chang Y.-A. (2014). Bacterial communities in semen from men of infertile couples: Metagenomic sequencing reveals relationships of seminal microbiota to semen quality. PLoS ONE.

[45] Mändar R., Punab M., Korrovits P., Türk S., Ausmees K., Lapp E., Preem J.-K., Oopkaup K., Salumets A., Truu J. (2017). Seminal microbiome in men with and without prostatitis. Int. J. Urol..

[46] Toscano M., De Grandi R., Stronati L., De Vecchi E., Drago L. (2017). Effect of *Lactobacillus rhamnosus* HN001 and *Bifidobacterium longum* BB536 on the healthy gut microbiota composition at phyla and species level: A preliminary study. World J. Gastroenterol..

[47] Khailova L., Petrie B., Baird C.H., Dominguez Rieg J.A., Wischmeyer P.E. (2014). *Lactobacillus rhamnosus* GG and *Bifidobacterium longum* attenuate lung injury and inflammatory response in experimental sepsis. PLoS ONE.

[48] Inturri R., Stivala A., Furneri P.M., Blandino G. (2016). Growth and adhesion to HT-29 cells inhibition of Gram-negatives by *Bifidobacterium longum* BB536 e *Lactobacillus rhamnosus* HN001 alone and in combination. Eur. Rev. Med. Pharmacol. Sci..

[49] Inatomi T., Otomaru K. (2018). Effect of dietary probiotics on the semen traits and antioxidative activity of male broiler breeders. Sci. Rep..

[50] Dardmeh F., Alipour H., Gazerani P., van der Horst G., Brandsborg E., Nielsen H.I. (2017). *Lactobacillus rhamnosus* PB01 (DSM 14870) supplementation affects markers of sperm kinematic parameters in a diet-induced obesity mice model. PLoS ONE.

[51] Tremellen K. (2016). Gut Endotoxin Leading to a Decline IN Gonadal function (GELDING)—A novel theory for the development of late onset hypogonadism in obese men. Basic Clin. Androl..

[52] Tremellen K., Pearce K. (2017). Probiotics to improve testicular function (Andrology 5:439-444, 2017)—A comment on mechanism of action and therapeutic potential of probiotics beyond reproduction. Andrology.

[53] Levin E.D., Bencan Z., Cerutti D.T. (2007). Anxiolytic effects of nicotine in zebrafish. Physiol. Behav..

[54] Stewart A.M., Braubach O., Spitsbergen J., Gerlai R., Kalueff A.V. (2014). Zebrafish models for translational neuroscience research: From tank to bedside. Trends Neurosci..

[55] Cachat J., Stewart A., Grossman L., Gaikwad S., Kadri F., Chung K.M., Wu N., Wong K., Roy S., Suciu C. (2010). Measuring behavioral and endocrine responses to novelty stress in adult zebrafish. Nat. Protoc..

[56] Davis D.J., Bryda E.C., Gillespie C.H., Ericsson A.C. (2016). Microbial modulation of behavior and stress responses in zebrafish larvae. Behav. Brain Res..

[57] Sampson T.R., Mazmanian S.K. (2015). Control of Brain Development, Function, and Behavior by the Microbiome. Cell Host Microbe.

[58] Bravo J.A., Forsythe P., Chew M.V., Escaravage E., Savignac H.M., Dinan T.G., Bienenstock J., Cryan J.F. (2011). Ingestion of *Lactobacillus* strain regulates emotional behavior and central GABA receptor expression in a mouse via the vagus nerve. Proc. Natl. Acad. Sci. USA.

[59] Messaoudi M., Lalonde R., Violle N., Javelot H., Desor D., Nejdi A., Bisson J.-F., Rougeot C., Pichelin M., Cazaubiel M. (2011). Assessment of psychotropic-like properties of a probiotic formulation (*Lactobacillus helveticus* R0052 and *Bifidobacterium longum* R0175) in rats and human subjects. Br. J. Nutr..

[60] Savignac H.M., Kiely B., Dinan T.G., Cryan J.F. (2014). Bifidobacteria exert strain-specific effects on stress-related behavior and physiology in BALB/c mice. Neurogastroenterol. Motil..

[61] Savignac H.M., Tramullas M., Kiely B., Dinan T.G., Cryan J.F. (2015). Bifidobacteria modulate cognitive processes in an anxious mouse strain. Behav. Brain Res..

