# Supplementary material for: Diet Supplemented with Antioxidant and Anti-Inflammatory Probiotics Improves Sperm Quality after Only One Spermatogenic Cycle in Zebrafish Model
Source: Nutrients. 2019 Apr 13;11(4):843. doi: 10.3390/nu11040843 (PMC6549425; doi:10.3390/nu11040843)

Table S1. Weight values (mean ± s.e.) for the groups before and after treatment.

	**Weight (g)**
Control group	
Day 0	0.3070 ± 0.03297
Day 21	0.3184 ± 0.03290
Maltodextrin group	
Day 0	0.2593 ± 0.02577
Day 21	0.2872 ± 0.02705
Probiotic group	
Day 0	0.3249 ± 0.04026
Day 21	0.3489 ± 0.04043

Table S2. Sperm quality values (mean ± s.e.) for the groups before and after treatment.

	**Concentration (10^8 cells/mL)**	**Total motility (%)**	**Progressive motility (%)**	**Slow cells (%)**	**Medium cells (%)**	**Fast cells (%)**
Control group						
Day 0	36.21 ± 12.19	26.44 ± 6.528	14.33 ± 3.979	6.67 ± 1.953	9.250 ± 2.094	12.00 ± 2.464
Day 21	30.19 ± 10.15	29.97 ± 6.194	15.13 ± 5.534	5.500 ± 1.180	9.625 ± 3.610	10.38 ± 4.071
Maltodextrin group						
Day 0	10.29 ± 3.015	24.56 ± 5.528	11.63 ± 3.679	5.286 ± 1.248	8.286 ± 1.960	12.43 ± 3.100
Day 21	41.57 ± 18.16	24.97 ± 6.766	11.00 ± 3.964	4.875 ± 1.457	8.500 ± 2.368	8.625 ± 3.082
Probiotic group						
Day 0	44.58 ± 16.40	28.39 ± 6.463	15.22 ± 4.713	5.444 ± 1.617	9.778 ± 2.924	12.44 ± 3.594
Day 21	110.10 ± 23.13# **	48.36 ± 7.319¥ **	22.73 ± 5.092¥ **	9.455 ± 1.729¥ *	14.82 ± 2.860¥ *	19.82 ± 4.018¥ *

#Wilcoxon test for paired samples; ¥t-Student test for paired samples; (*) $p<0.050$; (**) $p<0.010$.

Table S3. Novel tank values for behaviour estimators (mean ± s.e.) for the groups before and after treatment.

	N. of crossings	Scores in the top (%)
Control group		
Day 0	14.58 ± 1.909	44.94 ± 8.674
Day 21	17.00 ± 3.389	53.05 ± 8.521
Maltodextrin group		
Day 0	10.00 ± 2.532	51.07 ± 10.93
Day 21	11.50 ± 2.626	28.38 ± 8.157
Probiotic group		
Day 0	8.417 ± 2.006	43.99 ± 10.73
Day 21	17.67 ± 3.467 ¥ *	48.24 ± 8.373

¥t-Student test for paired samples; (*) $p<0.050$

Figure S1. Before-after graphs for (**A**) Concentration, (**B**) total motility and (**C**) progressive motility at 0 and 21 days obtained for each experimental group. "M" and "P" refers to the experimental groups: control and maltodextrin respectively. "M#" indicates the number of the male.

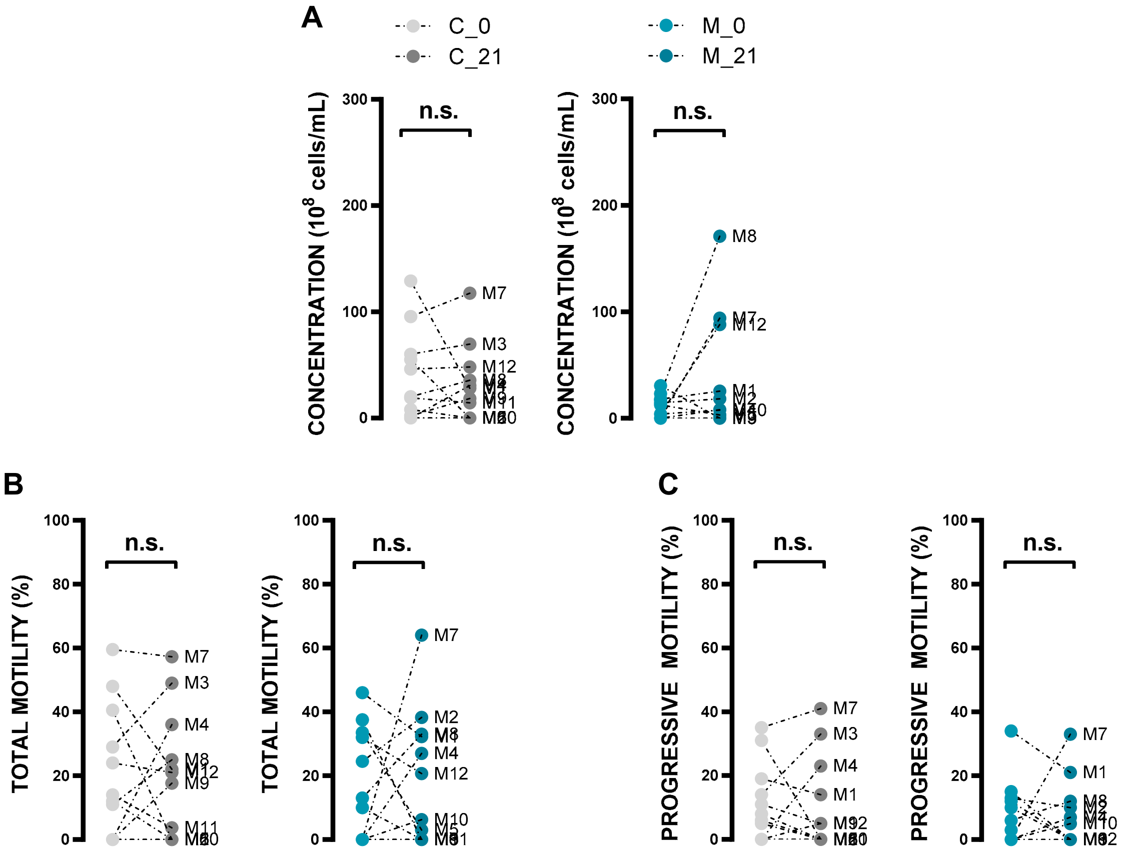

Figure S2. (**A**) PCA analysis for Computer Assisted Sperm Analysis (CASA) variables for probiotic-fed group. (**B**) Representation of the experimental group in a principal component plane.

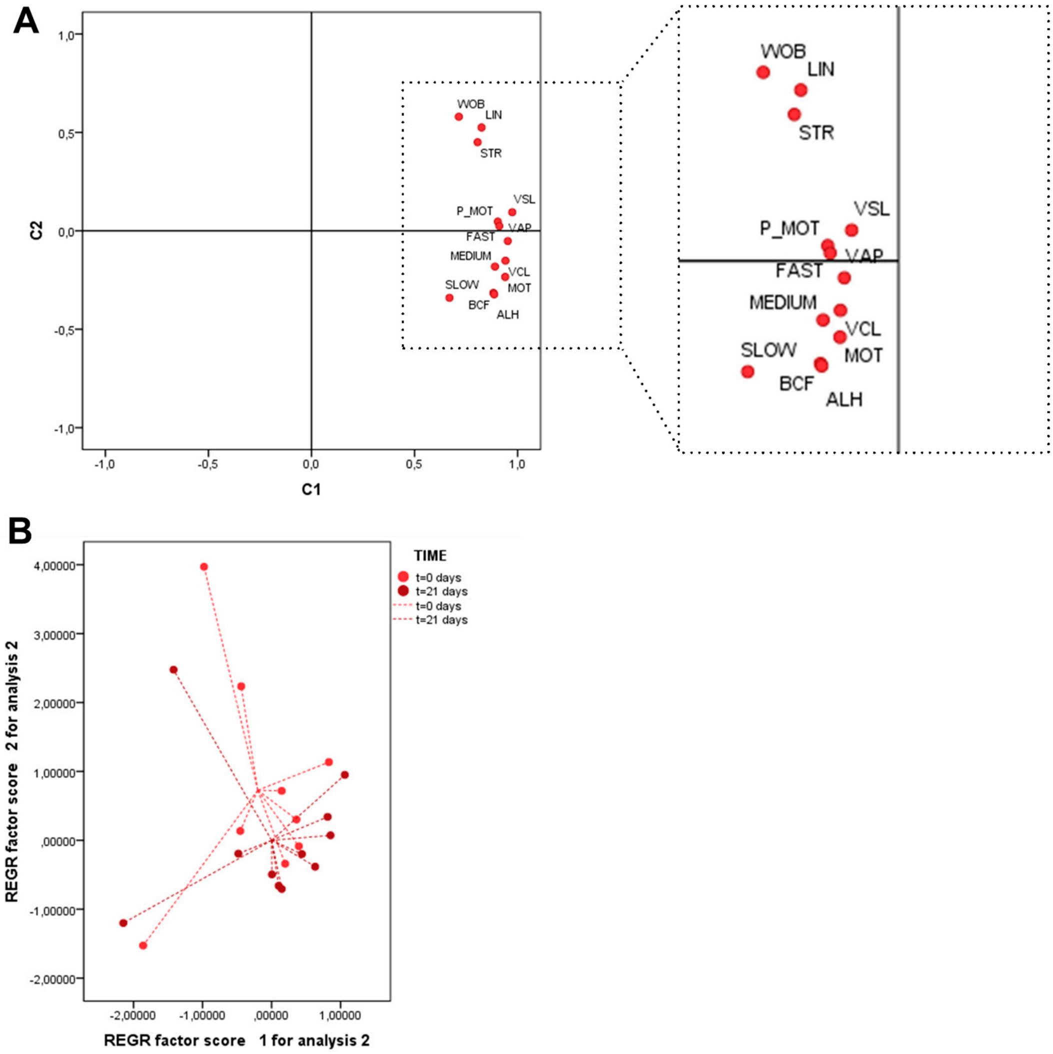

Supplement: Supplementary file 1 [file nutrients-11-00843-s001.pdf]
